# Supplementary material for: Implementation of mass drug administration for neglected tropical diseases in Guinea during the COVID-19 pandemic
Source: PLoS Negl Trop Dis. 2021 Sep 27;15(9):e0009807. doi: 10.1371/journal.pntd.0009807 (PMC8496865; doi:10.1371/journal.pntd.0009807)
Supplement: S1 Checklist — (DOCX) [file pntd.0009807.s001.docx]

**Supervision Checklist - COVID-19 Barrier Measures during MDA**

**Region:**

**District:**

**Health center:**

**Supervisor team:**

**Date:**

1. **COVID-19 epidemic:**

Is the district affected by COVID-19?

Yes

If yes

What is the number of cases? _________________

Is there ongoing community transmission?

Yes

No

No

Have supervisors been tested for COVID-19 before travelling for supervision?

Yes

If yes, did anyone test positive?

Yes

No

No

Is self-monitoring of COVID-19 symptoms done by MDA actors (Supervisors and CDDs) on a daily basis?

Yes

If yes, is self-monitoring reported to someone?

☐ Yes

☐ No

No

Were COVID-19 barrier measures covered during the training of CDDs?

Yes

No

Were COVID-19 preventive measures included in NTDs communication materials?

Yes

If yes, were the communication materials include e.g. in public criers, CDDs, radio messages?

Yes

No

No

1. **Hygiene measures**

Are CDDs washing their hand or using hand sanitizer before entry and after exit of houses compound?

Yes

No

Are beneficiaries washing their hands or using hand sanitizer before receiving treatment?

Yes

No

Are CDDs wearing gloves during MDA?

Yes

No

1. **Barrier measures**

Are the CDDs wearing a mask/facial covering during activities?

Yes

If yes, is the mask/facial covering being worn properly?

Yes

No

No

Are the beneficiaries wearing a mask/facial covering during the MDA?

Yes

No

Is the physical distancing (2 meters) being respected between CDDs and householder members during MDA?

Yes

No

Are people from other households following the CDDs?

Yes

No

Are people gathering around CDDs during MDA?

Yes

No

1. **MDA strategy**

Is door-to-door strategy being used in community-based treatments?

Yes

If yes, are only household members being treated together (without other people from another household)?

Yes

No

No

If no, how are you doing the MDA ___________________________________________________

_______________________________________________________________________________

Are students being treated outside the classroom in a school-based MDA?

Yes

No

Are beneficiaries touching the dose pole during their measurement?

Yes

If yes, is the dose pole washed or disinfected after each household?

Yes

No

No

If no, how is the dose pole being held? _______________________________________________

Do CDDs touch the hands of beneficiaries when handing over the tablets?

Yes

No

Were the CDDs reluctant to participate in the MDA due the COVID-19 context?

Yes

If yes, could you provide more information____________________________________________

No

Were the communities reluctant to participate in the MDA due the COVID-19 context?

Yes

If yes, could you provide more information___________________________________________________

No

Could you take photos and or make short videos of the MDA activities in COVID-19 context and share with partners and donors?

☐ Yes

☐ No

Any other thoughts or observations including logistics______________________

__________________________________________________________________________________________________________________________________________________________________________________________

Corrective measures put in place in case of weakness observed _________________________________________

_______________________________________________________________________________________________________________________________________________________________________________________________________________________________________________________________________________________
