# Supplementary material for: Implementation of mass drug administration for neglected tropical diseases in Guinea during the COVID-19 pandemic
Source: PLoS Negl Trop Dis. 2021 Sep 27;15(9):e0009807. doi: 10.1371/journal.pntd.0009807 (PMC8496865; doi:10.1371/journal.pntd.0009807)
Supplement: Attachment — Submitted filename: Responses to reviewer comments.docx [file pntd.0009807.s005.docx]

Matthew C Freeman, MPH, Ph.D.

Associate Editor

PLOS Neglected Tropical Diseases

Christine Petersen

Deputy Editor

PLOS Neglected Tropical Diseases

Dear Editors,

Thank you for your correspondence of June 18, 2021. We thank you for considering our paper and for your instructions. We would also like to thank four reviewers for their time and constructive comments and suggestions. We have now considered all the comments carefully and addressed them one by one. Our point-by-point responses are listed below this letter. We hope that this revised manuscript is now suitable for publication in the journal.

As requested, we have now uploaded the necessary documents for your consideration, including a clean version of the manuscript and a version with tracked changes. Please also note that we have followed your instructions as follows:

- Figure files: We have run the figures through PACE and these figures now meet PLOS requirements.
- Database Requirements: The database for the checklist data collected is uploaded as Supporting information S2. The MDA coverage data are already contained in the paper in Table 1.
- Reproducibility: The checklist used for the supervision is already uploaded Supporting information S1. There is no research protocol available as this was not a research project.
- References: Please refer to the responses to reviewers below. The references are now checked and edited one by one according to the journal requirements. Weblinks are provided where available. Non-eligible references are now removed. References are now renumbered. Please also provided a new reference (#14) in relation to the number of COVID-19 reported cases in Guinea.

We look forward to hearing from you.

King regards

Benoit Dembele

On behalf of all authors

**Point-by-point responses to Reviewers:**

**Responses to Reviewer 1:**

**Methods:**

Are the objectives of the study clearly articulated with a clear testable hypothesis stated? yes but no hypothesis du to study design(report) -Is the study design appropriate to address the stated objectives? Yes -Is the population clearly described and appropriate for the hypothesis being tested? Yes only population is clearly described -Is the sample size sufficient to ensure adequate power to address the hypothesis being tested? The sample signe like too small to be representative.

*Response: Thank you for the comments. Yes, we recognized that the sample size was relatively small. We have listed this as one of the limitations of the paper and have explained the situation in the Discussion.*

**Results:**

Does the analysis presented match the analysis plan? Yes -Are the results clearly and completely presented? Yes but should be more clear to compare MDA coverage with the results of previous MDA to measure the impact of COVID-19.

-Are the figures (Tables, Images) of sufficient quality for clarity? Yes bu need to be review with comment above

*Response: Thank you for the comments. The primary purpose of this paper was not to assess the impact COVID-19 on the MDA coverage but how the MDA was conducted during the COVID-19 pandemic with preventive and barrier measures. MDA coverage is influenced by multiple factors including COVID-19. Without controlling all other factors, it is difficult to attribute any impact on MDA coverage to COVID-19. The 2020 MDA was a national program activity and was not designed to control other factors for comparative analysis on COVID-19. Therefore, we decided not to conduct such a comparison.*

**Conclusions:**

Are the conclusions supported by the data presented? Yes -Are the limitations of analysis clearly described? Yes -Do the authors discuss how these data can be helpful to advance our understanding of the topic under study? Yes but not sufficient -Is public health relevance addressed? The study focuses on the proportions of actors who apply the barrier measures. It doesn't give much scientific interest. It would have been good to push the study by measuring the impact of the campaign on the occurrence of new cases of COVID-19 and especially if actors have developed signs after this campaign.

*Response: Thank you for the comments. This paper describes the experience of Guinea successfully implementing the MDA during the unprecedented COVID-19 pandemic. Such experience may not have scientific interest but does have major programmatic interest. The MDA activity was a routine national program activity and was not designed to measure the impact on COVID-19 transmission. However, we have now added a paragraph and discussed this point in the Discussion. We cited the published data on new case reporting in Guinea for the 5 weeks before, during and after MDA, which did not show any increasing trend in new case reporting in Guinea, instead the national trend of new case reporting was downward after the MDA. We did not follow CDDs on COVID-19 infection after MDA but any new infection should have been reflected in the national new cases reported. None of the supervisors who returned to Conakry reported COVID-19 infection or being tested positive.*

**Summary and General Comments:**

The study is interesting but remains in the form of a report. The authors could have extended this study by taking into account the impact of the implementation of barrier measures on the transmission of COVID-19. If they were effective, the results could be used as evidence to be strongly recommended to other countries.

*Response: Thank you for the comments. Please refer to the response above. We have now added this point in the Discussion.*

**Responses to Reviewer 2**

**Methods:**

The authors need to better explain the following in the section of risk assessment “drug distribution platforms”

*Response: The types of platforms were added to the text. The platforms normally used for MDA included community-based MDA with door to door or fixed-point distribution, and school-based MDA. The risk for each of these was assessed. The national guidance recommended avoiding grouping people and the community-based MDA with door-to-door strategy provided best way to avoid people grouping and was therefore selected for the MDA at the time.*

Please the following need more precision “travelers from Conakry avoid crowded vehicles.”

*Response: Precision added to the text*

Please the following is a repetition and not well written “The MDA and prevention and protection against COVID-19 messages were disseminated by local media before and during the campaign.

*Response: This sentence is removed. The relevant parts of previous sentences are revised.*

Please rewrite the following part in social mobilization section “Some routine activities, such as the national campaign launch ceremony were cancelled to prevent the gathering of crowds.”

*Response: The sentence is now edited.*

“Central level program staff and supervisors were tested for COVID-19 before leaving Conakry and only staff with negative results travelled to the districts and conducted the supervision. All supervisors observed COVID-19 mitigation measures during supervision visits, including wearing face masks, social distancing of two meters, and using hand sanitizer.”

*Response: Texts quoted are now edited. The corresponding paragraph is reorganized in conjunction with the next comment.*

As a limit the authors did not indicated whether those negative while in the field respected confinement period before working because the newly infected will not be positive directly I will suggest to the authors to add study population section and sample size for more clarification

*Response: Thanks for the comments and suggestion. The confinement period was not observed as this was not required at the time, but the COVID-19 preventive and barrier measures were respected, and symptoms were monitored on a daily basis when supervisors were in the field. A new Section “Study population and sampling” was added as suggested. As a result, the Sections “Supervision and Checklist” and the overview part of Results we reorganized. Hope this will provide a better flow of the paper.*

**Results:**

Yes the results are well presented “Thus, the results presented here relate to adherence to the contingency plan for the implementation of MDA in the context of the COVID-19 epidemic (barrier and preventive measures against COVID-19) in 14 HDs.” Read this part carefully something is missing”

Tables and figures are good

*Response: The sentence grammatically is correct and complete, but it is now simplified. As a result of the suggestion for a new section above, this overview part is reorganized and moved to the relevant parts in the Methods.*

**Conclusions:**

“The results demonstrate that implementation of MDA in the context of COVID-19 is programmatically challenging, but that with appropriate planning, resources, and training, risk mitigation measures can be rapidly applied.” I will suggest to remove “that”

*Response: Thanks. The second “that” is removed.*

**Summary and General Comments:**

Please revise this section size “The participatory approach, led by the NTDP, to develop the contingency plan contributed to wide acceptance of restarting the MDA from all key stakeholders.”

*Response: We were not sure what was requested here. But in conjunction with a comment from Reviewer 3, details on key stakeholders are added in the text. Thanks.*

Please read this again not sure if this statement is correct as it is “and it was not possible to calculate STH coverage for SAC only” . my question is why not even if it was integrated normally I will be possible to report

*Response: We agree that it should be possible to report separate coverage for STH in SAC, however this was not reported by the program, so we were not able to show this separately. The sentence is revised.*

This need also to be taken into account in the mitigation plan like asking and get agreement with communities for the best tie during the day distribute the drugs “During the rainy season, access to the target population and some places are difficult and people are not often in the household during the MDA campaign.”

*Response: thanks for the comments. We agree this would be ideal, but such challenges remain to the national programs after many years of intervention. This would be something to address in future MDAs.*

**Responses to Reviewer 3**

**Methods:**

The objectives should be clearly articulated, preferably by stating the general objective and 2-3 specific objectives. These should be closely aligned to Similarly clear study questions. The discussion should then be restructured to speak to the objectives.

*Response: We have added objectives to the last paragraph in the Introduction.*

**Summary and General Comments:**

The study is relevant to the current realities of COVID-19 and provides useful insights on how programs can implement interventions to safeguard gains already made, while taking necessary precautions to minimize the risk of getting infected with the disease.

*Response: Thanks for the comments.*

**Responses to Reviewer 4**

**Methods:**

In the 1st sentence of the Methods section, the word “in” seems to be a typo, please double check it

*Response: Thanks. You are right. This is removed.*

The break times are the riskiest periods during the training sessions, was there specific directions for these periods? If yes, please specify them

*Response: Yes absolutely, to mitigate that, refreshments were not provided to the group during the break for CDD training sessions. Every participant was given allowance instead. This is now added to the Training section.*

**Results:**

In table 1, please explain the coverage > 100% or discuss them in the appropriate section

*Response: Thanks for the comment. As we described in the Data analysis, we used the number of SAC as the denominator which was estimated using the national average proportion of SAC in the projected whole population according to the 2017 national census data. Such estimated numbers of SAC do not always represent the true numbers of SAC in communities at the time of MDA. It is very common to see either very low or very high (>100%) coverage rates in such a situation.*

Please check this sentence of the last paragraph of the Results section « Adherence to the mitigation measures for districts reporting COVID-19 cases was similar compared to districts where no case of COVID-19 was confirmed (Table 2). »

*Response: The sentence is now revised. “Adherence to the mitigation measures in districts where COVID-19 cases were reported was similar to that in districts where no case of COVID-19 was reported (Table 2).”*

The last sentence of the Results sections contains a word that need to be checked and replaced; the same sentence seems to consider a p value =0.05 as statistically significant. A p value should be <0.05 to be considered statistically significant.

*Response: Thanks for the observation. The word “reluctance” should have been “reluctant”. Yes, a p value =0.05 was considered statistically significant, as it is general that p≤0.05 is considered statistically significant. However this sentence is now removed due to the correction of the p-values and the change in the next comment.*

Some of the small numbers in the last table seems to require a Fisher Exact test instead of an uncorrected Chi square test (i.e. Household participants reluctant to participate in MDA line), Please double check

*Response: Tests were reviewed, and Fisher’s exact test is applied where required. P-values are now corrected, all showing >0.05.*

**Editorial and Data Presentation Modifications:**

Abstract:

A definition of the world CDDs team and Households would be helpful in understanding these concepts.

*Response: This is added – (two CDDs per team), but definition of household in Abstract would be too long. We have added this in the Methods when household is first mentioned.*

At least 2 sentences started with numbers

*Response: Such sentences are now revised to avoid starting with numbers.*

Background

-	Please add a reference at the end of the first paragraph of the Background section

*Response: This is added.*

-	Please split the last sentence of the 3rd paragraph into two sentences to gain in clarity

*Response: Thanks, and this is done.*

Discussion

-	At the end of the second paragraph of the Discussion sections, it would be informative to name the key stakeholders evocated.

*Response: detail information provided.*

-	Please check the 2nd sentence of the 4th paragraph “Firstly, six out of seven HDs of LF districts which did not achieve the minimum coverage had reported cases of COVID-19. » for clarity

*Response: Thanks for pointing this out. It was an oversight. It should have been eight out of 9 HDs… and this have now been corrected.*

-	In the same paragraph and elsewhere in the manuscript, the authors seem to be using interchangeably « households » and « households’ members », please look at that carefully and adapt according to the meaning of the concept in each sentence throughout the manuscript.

*Response: Thanks for the observation. We have now gone through the paper and changed “household members” to “household participants” which is more appropriate in the context.*

References

-	Many references used are not eligible for a peer reviewed paper

-	References that are not peer reviewed journal articles should have a web link and date of access

-	The types of documents eligible for citation as a reference should be check on the Journal’s website

*Response: The references are now checked and edited one by one according to the journal requirements. Weblinks are provided where available. Non-eligible references are now removed. References are now renumbered. Please also provided a new reference (#14) in relation to the number of COVID-19 reported cases in Guinea.*

**Summary and General Comments:**

This paper describes a real and current issue that threatened interventions targeting NTDs. It provides with an attitude that is well described and could help in the future when similar situation is faced.

*Response: Thanks for the comments.*

**Responses to additional comments on the manuscript (not sure from which reviewer)**

Can you demonstrate but after the MDA none increase of cases appear in the MDA zone and have assess the actors (supervisors and CDDs) to how many of them have presented COVID-19 signs after MDA.

*Response: This is similar comment to one of the Reviewer 1’s comments. Please refer to our response above to such a comment. We did not specifically follow this up in CDDs ad supervisors. But none of supervisors reported back a COVID-19 symptoms or being tested positive afterwards.*

How many CDDs were engaged in this campaign? 120 is representative?

*Response: A large number of CDDs (over 8000) participated in MDA across the 19 districts. The routine national MDA supervision only covers a small proportion od CDDs. We recognized that the sample size was relatively small have and listed this as one of the limitations of the paper in the Discussion. The results may not represent the true situation in the field.*

Evaluating a CDDS team only once during a campaign does not mean that CDDs scrupulously apply barrier measures every day.

*Response: Yes, CDDs may or may not follow the rules everyday, but this is a routine national supervision of the MDA activity, and it was not designed for detailed research purpose. We do not have such data to comment.*

As it is not the first MDA, it should be interesting to compare MDA coverage with the results of previous MDA to measure the impact of COVID-19.

*Response: Thank you for the comments. The primary purpose of this paper was not to assess the impact COVID-19 on the MDA coverage but how the MDA was conducted during the COVID-19 pandemic with preventive and barrier measures. MDA coverage is influenced by multiple factors including COVID-19. Without controlling all other factors, it is difficult to attribute any impact on MDA coverage to COVID-19. The 2020 MDA was a national program activity and was not designed to control other factors for comparative analysis on COVID-19. Therefore, we decided not to conduct such a comparison.*

Are the CDDs are informed that the supervisors are evaluate their actions?

*Response: No, they were not informed of their selection of supervision prior to the supervisors’ visit. This is now made clear in the Sampling section.*

To be a good paper authors should show the COVID-19 trend after the MDA campaign and demonstrate if some actors develop COVID-19 signs after MDA campaign.

*Response: Please see the response to the first comment in this section.*
